# The role of delayed cytoreductive nephrectomy following axitinib–toripalimab for the mRCC patient in current immunotherapy era: a case report and literature review

**DOI:** 10.3389/fimmu.2026.1755688

**Published:** 2026-03-11

**Authors:** Quan Zhou, Junlong Wang, Ma WenJing, Ye ZeLin, Jiasheng Yan, Xiaodong Jin

**Affiliations:** 1 Department of Urology, The First Affiliated Hospital of Zhejiang Chinese Medical University (Zhejiang Provincial Hospital of Chinese Medicine), Hangzhou, Zhejiang, China; 2 Zhejiang Chinese Medical University, Hangzhou, Zhejiang, China

**Keywords:** axitinib, case report, deferred cytoreductive nephrectomy, neoadjuvant therapy, renal cell carcinoma, toripalimab

## Abstract

The global incidence of renal cell carcinoma (RCC) has shown a significant upward trend in recent years, with growing health inequities related to its incidence. RCC remains the most lethal urological cancer. Although with the gradual popularization of physical examination screening, an increasing number of RCC patients have been detected and treated at an early stage, approximately 30% of patients are still diagnosed with locally advanced or metastatic RCC at the time of initial diagnosis. The treatment landscape for advanced or metastatic RCC has evolved substantially with the introduction of immune checkpoint inhibitors (ICIs) combined with tyrosine kinase inhibitors (TKIs). Neoadjuvant ICI–TKI combinations may downstage primary tumors and enable deferred cytoreductive nephrectomy (CN), but evidence regarding feasibility, efficacy, and safety remains limited. We present the case of a 50-year-old man with advanced clear-cell RCC and pulmonary metastases who received three cycles of neoadjuvant axitinib plus toripalimab. The regimen resulted in marked tumor shrinkage and necrosis, allowing safe robotic-assisted deferred CN. Pathology revealed extensive necrosis with minimal viable tumor. The treatment was overall well tolerated, and the patient recovered without recurrence. This case supports the feasibility and potential efficacy of neoadjuvant axitinib plus toripalimab followed by delayed CN in selected patients. Further studies are warranted to validate this sequential strategy and optimize perioperative safety management.

## Introduction

Renal cell carcinoma (RCC) accounts for approximately 3% of all adult malignancies (1). From 1992 to 2021, the global age-standardized incidence rate of kidney cancer increased significantly. In 2021, its disability-adjusted life year rate rose steadily with advancing age. The global population of individuals aged 65 and older is projected to reach 1.6 billion by 2050. Given that RCC is more common in the elderly, the burden on the healthcare system is also increasing. Additionally, from 1990 to 2021, the social inequality index increased while the concentration index decreased slightly, indicating that sociodemographic index-related health disparities in kidney cancer have intensified, and high-SDI countries remain the primary regions with concentrated kidney cancer burden. Against this, nearly one-third of patients have developed metastatic lesions at the time of diagnosis, and approximately 20%-40% of patients treated for localized RCC will eventually develop distant metastases (2).

While localized early-stage RCC can be managed surgically, locally advanced or metastatic RCC (aRCC/mRCC) are often inoperable due to tumor progression, dissemination, and metastasis. Furthermore, traditional radiotherapy and chemotherapy yield poor results, leading to an unfavorable prognosis and significantly threatening patient survival (3). Over the past two decades, the first-line treatment landscape for aRCC/mRCC has undergone a profound transformation, evolving from cytokine-based therapies to vascular endothelial growth factor receptor (VEGFR)-targeted agents, and more recently, to immune checkpoint inhibitors (ICIs) and their combinations with tyrosine kinase inhibitors (TKIs) (4). Angiogenesis is a key driver of solid tumor growth and invasion, and antiangiogenic therapy has become an established strategy across multiple malignancies, providing a mechanistic rationale for VEGFR-TKI–based neoadjuvant downsizing (5). Immune checkpoint blockade and emerging co-inhibitory targets, together with key oncogenic signaling pathways linked to immune evasion, provide a biological rationale for combination immunotherapy strategies in RCC (6). Among these, axitinib, a second-generation anti-angiogenic targeted agent, is a potent and selective TKI of VEGFR-1, -2, and -3. It has been approved by global health authorities for the treatment of aRCC, both as monotherapy in the second-line setting and in combination with avelumab or pembrolizumab for first-line treatment (7). Toripalimab is a humanized anti-programmed cell death protein-1 (PD-1) IgG4k monoclonal antibody. Notably, the RENOTORCH study investigating axitinib plus toripalimab—the first successful trial of targeted-immunotherapy combination in Chinese intermediate-/poor-risk RCC patients—demonstrated clinical efficacy that was non-inferior or even superior to previous studies, although overall survival data remain immature (8). Additionally, patients with aRCC/mRCC may still derive benefit from surgery. CN was proposed to improve antitumor immune system response by reducing the burden of RCC that was producing factors interfering with T-cell function (9). While upfront cytoreductive nephrectomy (CN) is no longer considered the standard of care for intermediate-risk, asymptomatic patients with primary clear cell RCC(ccRCC) and all poor-risk, asymptomatic patients with metastatic disease, retrospective data from the International Metastatic RCC Database Consortium (IMDC) suggest that cytoreductive nephrectomy retains clinical value in patients receiving vascular endothelial growth factor (VEGF)-targeted therapy (4, 9). The efficacy of novel systemic therapies is challenging standard management approaches for some patients with metastatic disease.

Herein, we report a case of advanced ccRCC in which neoadjuvant toripalimab plus axitinib enabled deferred cytoreductive nephrectomy. A literature search of PubMed, Embase, and Web of Science (through January 2026) did not identify previous case reports describing this exact sequence in aRCC/mRCC.

## Case presentation

A 50-year-old male presented with a six-month history of fatigue without an obvious cause and an approximate 5 kg weight loss over the same period. In May 2025, the patient self-palpated a mass in his left abdomen, prompting imaging studies. An abdominal CT scan performed on May 16, 2025, revealed a large space-occupying lesion in the left kidney, measuring approximately 107×135×133 mm, highly suggestive of renal malignancy. A PET-CT scan on May 19, 2025, confirmed a large mass in the left renal region with significantly increased FDG metabolism, alongside multiple bilateral pulmonary nodules, some with increased metabolism, indicating pulmonary metastases. A percutaneous needle biopsy was performed on May 20, 2025. Pathological examination confirmed a malignant tumor, and immunohistochemistry (IHC) supported the diagnosis of ccRCC. IHC results were as follows: P53(5%+),Ki-67(5%+),PAX-8(+), CAIX(+), CD10(+), EMA(+), E-cad (+), Vim (+), P504S(+), TFE3(+). There was no significant family history. The patient was clinically assessed as belonging to the IMDC high-risk group and the MSKCC high-risk group (10).

Beginning on May 27, 2025, we established a neoadjuvant therapy regimen of “axitinib (5mg, orally, twice daily) in combination with toripalimab (160mg, intravenous infusion, every 3 weeks)” for the patient and obtained the patient’s consent. Following initial treatment, laboratory tests showed a hemoglobin level of 82 g/L and a high-sensitivity C-reactive protein (hs-CRP) level of 107.70 mg/L. On June 10, 2025, the patient was admitted to the hospital for review due to abdominal pain. CT urography (CTU) and renal artery CTA revealed a large solid mass in the left kidney (approximately 147×125×133 mm) with markedly heterogeneous enhancement and destruction of some calyces; a complete blood count showed elevated white blood cells, indicating a significant inflammatory response. After receiving anti-infective and nutritional support therapy, the patient underwent the second cycle of immunotherapy on June 16. Post-treatment, hemoglobin was 97 g/L, and hs-CRP had decreased to 68.33 mg/L.

The third cycle of immunotherapy was completed on July 16, 2025. During this period, the lowest recorded hemoglobin was 75 g/L, and hs-CRP was 68.01 mg/L. The patient experienced intermittent fever and mild anemia, which resolved after symptomatic and supportive treatment.

On August 1, 2025, the patient was admitted due to left lumbar pain and fatigue, with hemoglobin dropping to 63 g/L. Following a hematology consultation, the axitinib and toripalimab treatment was temporarily held, and subcutaneous erythropoietin was administered. On August 21, the patient was readmitted with fever (peak temperature 39 °C), which improved after anti-infective treatment. A follow-up contrast-enhanced CT scan on August 26, 2025, demonstrated significant shrinkage of the left renal mass (approximately 90×77×98 mm). Gas shadows were visible within the tumor, suggesting tumor necrosis, and the surrounding inflammatory changes had resolved. Radiographic response to neoadjuvant therapy was assessed using RECIST 1.1 criteria (11). The primary renal mass was selected as the target lesion (pulmonary nodules were not measurable). The longest diameter decreased from 135 mm at baseline (May 16, 2025) to 98 mm on the preoperative CT (August 26, 2025), corresponding to a 27.4% reduction and meeting criteria for stable disease (SD). Notably, intratumoral gas and liquefactive necrosis were observed on imaging. After evaluation by a multidisciplinary team (MDT) and upon improvement of the patient’s general condition, he underwent robot-assisted laparoscopic radical left nephrectomy on September 2, 2025. During the operation, it was found that the renal tumor protruded significantly and was closely adherent to the left colon, lateral peritoneum, and spleen. During the separation process, leakage of intestinal contents occurred. Considering the possibility of renal cancer invading the intestine, a portion of the colonic tissue suspected to be invaded by the tumor was resected and sent for pathological examination. Subsequently, a partial colectomy with enterorrhaphy was performed (before this operation, the patient had completed intestinal cleaning by taking polyethylene glycol powder orally, so a primary intestinal anastomosis was performed after resecting part of the intestine).The surgery was completed successfully with a bleeding volume of approximately 200 milliliters, and one left renal fossa drainage tube was indwelled.

Postoperative pathology confirmed ccRCC (WHO/ISUP Grade 3) with treatment-related changes. The tumor measured approximately 11.5×7.5×6.5 cm, predominantly composed of necrotic tissue with only a small residual component of viable tumor cells. No vascular or perineural invasion was identified, and the renal sinus fat was uninvolved. Tumor invasion was present in the renal pelvis, calyces, and renal capsule. The ureteral margin was free of carcinoma. None of the 5 resected hilar lymph nodes contained metastasis (0/5). Immunohistochemistry results included: P53 (scattered positive/wild-type expression), Ki-67 (10% positive), CK7(-), CK20(-), CK34βE12(-), E-cad (+), EMA(+), CD10(+), CAIX(+), PAX-8(+), CD117(-), P504S(+), SDHB (+/no loss), Vim (+), TFE3 (focally weak positive), CD68 (histiocytes+), CD163 (histiocytes+), ALK(-), PAX-2(+), Oct3/4(-), P63(-). These findings indicated low proliferative activity in the residual tumor cells post-treatment. The submitted “colonic tissue” showed no carcinoma, consisting instead of fibroadipose and smooth muscle tissue with histiocytic reaction.

On September 20, 2025, the patient developed fat liquefaction at the surgical incision site, accompanied by mild exudate. A CT scan showed local exudation and gas shadows in the operative area, with blurring of the peritoneal planes. The condition improved after wound cleansing and dressing changes, anti-infective therapy, and nutritional support. The patient’s hemoglobin level is 117g/L, and the high-sensitivity C-reactive protein level is 55.34mg/L.

On October 16, 2025, the patient’s rechecked the high-sensitivity C-reactive protein level was 6.36mg/L.

On November 21, 2025, the patient was hospitalized to continue the postoperative adjuvant therapy of “axitinib (5mg, oral administration, twice a day) combined with Toripalimab (160mg, intravenous drip, once every 3 weeks)”. During this period, a rechecked PET-CT indicated low-metabolism small nodules in both lungs. Compared with the PET-CT image on May 17, 2025, the lesions were significantly reduced, some disappeared, and the metabolism was decreased, which was considered to be a significant inhibition of tumor activity after treatment.

**Figure 1:**
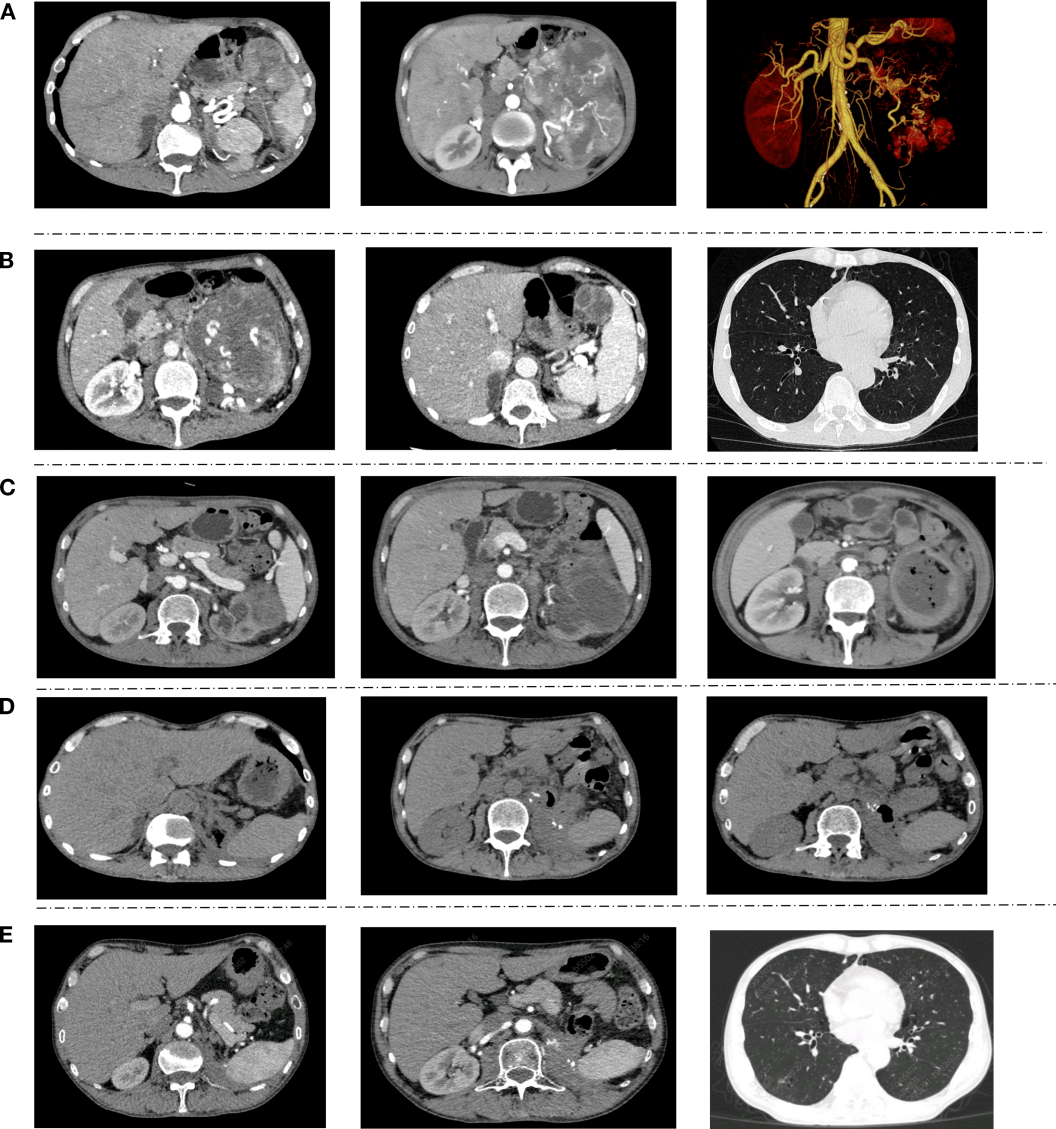
CT images at different time points: **(A)** Initial Presentation: A large left renal mass, measuring approximately 107 mm x 135 mm x 133 mm, is identified, highly suggestive of a malignant neoplasm, likely ccRCC. The left renal artery is tortuous, with its distal branches supplying the tumor. Splenomegaly is noted; **(B)** 3 Weeks Post-First Cycle of Immunotherapy and Targeted Therapy: The left renal mass shows no significant enlargement. The distal tumor-feeding branches of the renal artery exhibit mild attenuation. A left hilar mass is observed, with a larger cross-sectional dimension of approximately 17 mm x 19 mm; **(C)** Pre-operative, Post-Three Cycles of Combined Targeted and Immunotherapy: The left renal mass has significantly decreased in size compared to previous scans, now measuring approximately 90 mm x 77 mm x 98 mm. Internal liquefactive necrosis accompanied by gas shadows is evident within the mass; **(D)** 17 Days Post-operation: The left renal surgical bed shows multiple exudative changes with localized gas shadows. The local fat planes in the abdomen and pelvis appear blurred. A localized exudative change accompanied by gas shadows is seen in the subcutaneous incision site of the left abdomen. **(E)** Six weeks after the operation, a significant amount of absorption of the exudate in the surgical area of the left kidney can be observed. There are scattered small nodules in both lungs, which are roughly similar to the images before the operation.

**Figure 2:**
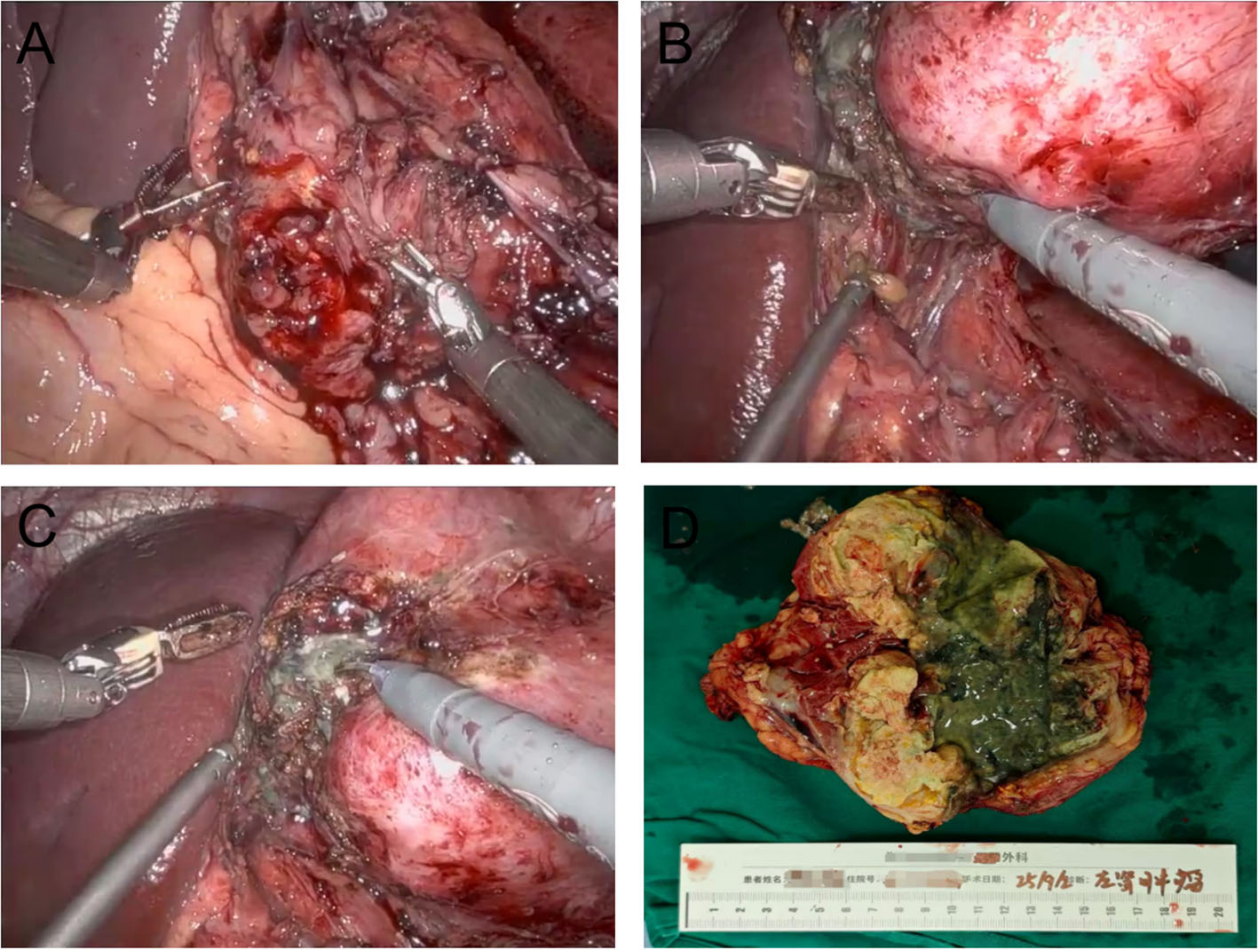
Intraoperative video screenshots and postoperative specimen overview: **(A-C)** The renal hilum was densely adherent to the mesentery. During dissection, gray-white enteric contents were observed leaking from the bowel; **(D)** The surgical specimen, comprising normal renal tissue and the massive renal tumor, is partially covered with dark green enteric contents.

**Figure 3:**
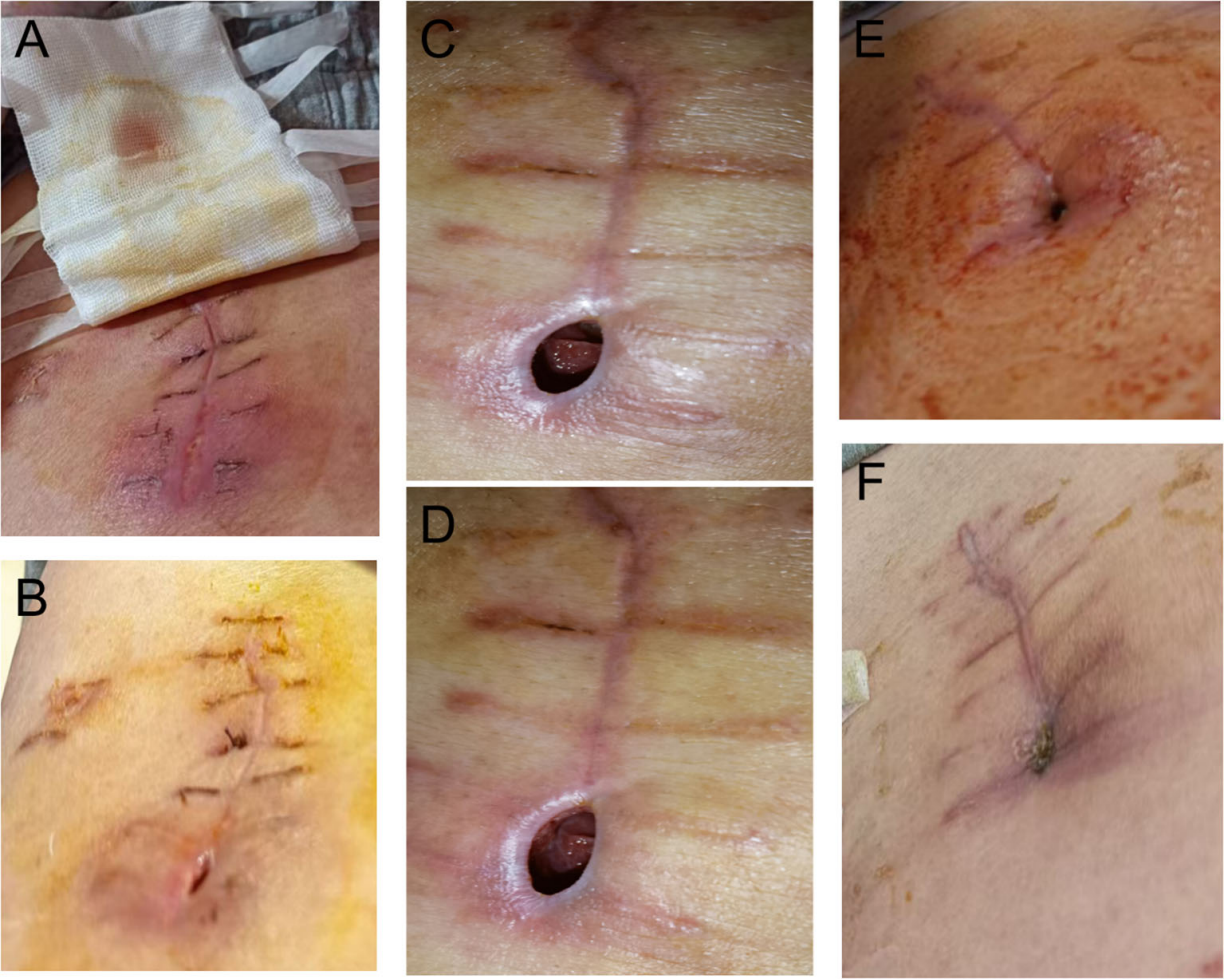
Photographic documentation of the surgical incision site during healing from fat liquefaction: **(A, B)** Postoperative Day 17: The wound exhibits yellowish exudate with no other subjective symptoms reported; **(C, D)** Day 11 of Wound Debridement and Dressing Changes: No further exudation of yellow fluid from the subcutaneous tissue is observed; **(E, F)** Week 3 of Debridement and Dressing Changes: The wound is nearly fully healed.

**Figure 4:**
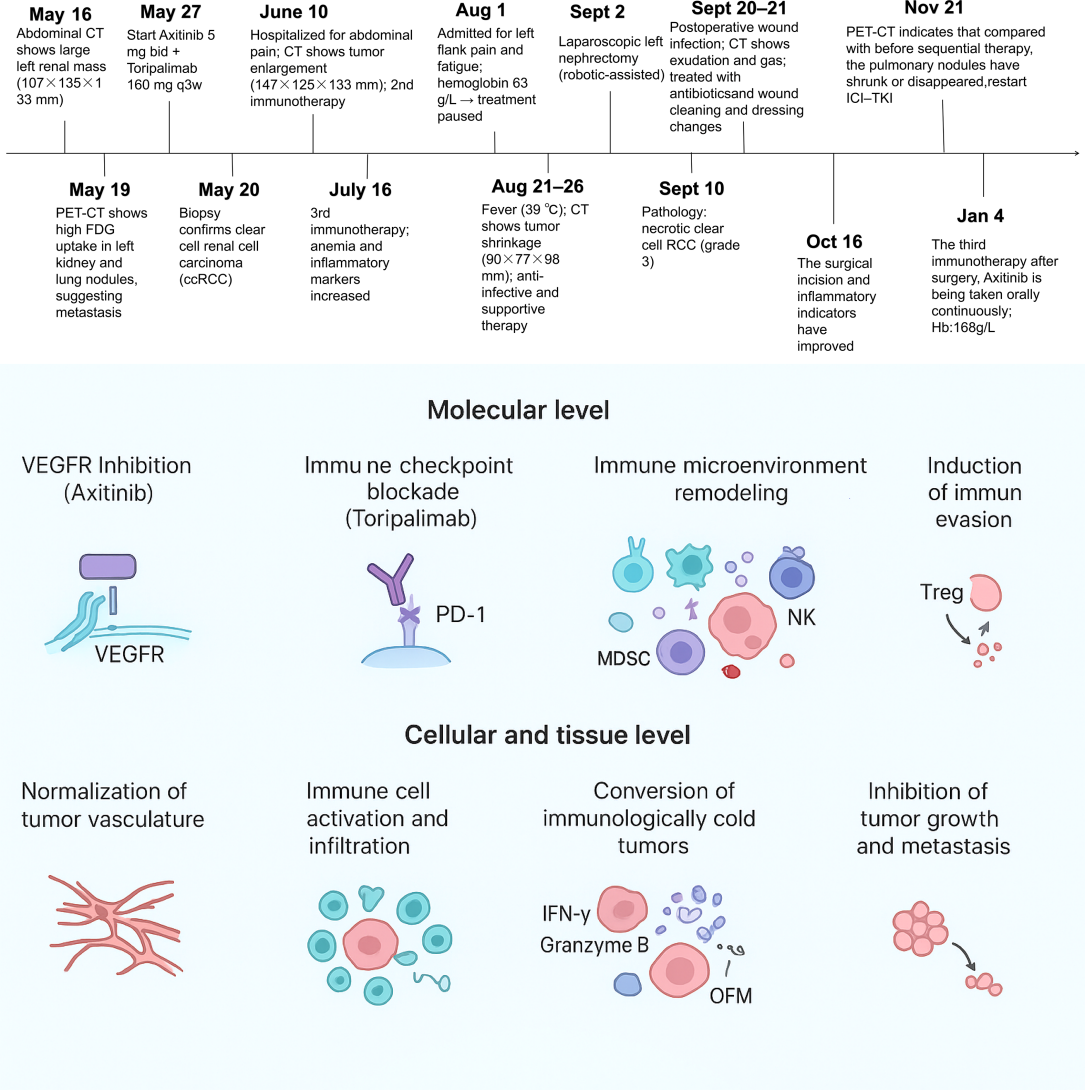
The timeline picture of this case.

Subsequently, the patient was hospitalized for the sequential treatment on December 9, 2025, and January 4, 2026, respectively. On January 3, 2026, the hemoglobin level was 168g/L. At the time of writing this article, the patient’s KPS score had increased from 70 at the onset of the disease to 100. Imaging examinations showed no recurrence or new metastases, and hemoglobin and platelets remained normal. Adjuvant therapy (axitinib + toripalimab) was resumed postoperatively and well-tolerated, with no grade ≥2 adverse events, further supporting sustained disease control potential. **Figure 1** shows CT images throughout the timeline, **Figure 2** includes intraoperative video screenshots and the postoperative specimen, **Figure 3** illustrates the changes associated with incision fat liquefaction, and **Figure 4** provides a timeline graphic.

## Discussion

In recent years, accumulating evidence indicates that the tumor microenvironment (TME) is a major determinant of antitumor immunity and immunotherapy outcomes, supporting the biological rationale for combining VEGFR inhibition with PD-1 blockade in RCC (12). A number of key international phase III studies (such as CLEAR, KEYNOTE-426 and other pivotal trials) have confirmed that the combined application of ICIs and TKIs has significantly improved outcomes for patients with aRCC/mRCC, establishing itself as a standard first-line treatment recommended by international and Chinese clinical guidelines (7, 8, 13–16). According to the latest NCCN (v1.2026) guidelines and Chinese expert consensus on the systemic treatment of aRCC/mRCC, axitinib combined with a PD-1 inhibitor is one of the preferred regimens for intermediate- and poor-risk patients, offering high objective response rates (ORR) and durable disease control (7, 17). However, none of these studies included the Chinese population, making the applicability of their results in Chinese clinical practice questionable. The phase III RENOTORCH study, published in 2024, fills this gap and is of landmark significance (8). As the first large-scale randomized trial conducted in a Chinese population, demonstrated that toripalimab plus axitinib significantly prolonged progression-free survival compared to sunitinib (median 18.0 months vs. 9.8 months; HR = 0.65) and achieved a higher ORR (55.7% vs. 29.6%). This treatment plan has been included in the latest edition of “GUIDELINES OF CHINESE SOCIETY OF CLINICAL ONCOLOGY (CSCO) KIDNEY CANCER” and has received a Level I recommendation (Category IA evidence) (13). It is expected to bring survival benefits to Chinese patients with aRCC/mRCC, especially those with intermediate and high-risk IMDC/MSKCC scores (18). Regarding safety, according to the previous studies (8, 14, 15), axitinib-based combination therapies have demonstrated a favorable and manageable safety profile while maintaining comparable efficacy benefits. Among them, according to the RENOTORCH study (8), toripalimab combined with axitinib exhibits a manageable toxicity profile. The most common adverse events include hypothyroidism (10.1%), hyperthyroidism (6.3%), rash (4.8%), reversible liver dysfunction (2.9%), diarrhea (2.9%), adrenal insufficiency, increased TSH, increased serum creatinine, and immune-mediated pneumonia (2.4% each), which is comparable to other TKI-PD-1 inhibitor combinations like pembrolizumab plus axitinib and avelumab plus axitinib (2, 19). In this case, after three cycles of toripalimab plus axitinib, the primary tumor, which was extensively compressing surrounding vessels and organs, showed significant shrinkage and extensive necrosis. No severe immune-related adverse events [particularly pneumonitis, hepatitis, colitis, and nephritis (20)] were observed in this case, enabling the patient to become eligible for surgery. Moreover, the patient no longer experiences recurrent fever, fatigue, low back pain, and other symptoms after the operation. Consistent with the Chinese consensus on the systemic treatment of aRCC/mRCC (17),This further corroborates the real-world efficacy and safety of this regimen in Chinese patients.

The success of immune-targeted combinations has also spurred growing interest in perioperative immunotherapy strategies. Neoadjuvant therapy prior to CN aims to reduce the primary tumor burden, lower surgical risk, and potentially induce systemic anti-tumor immune activation, which may improve long-term outcomes. Based on the sequence relative to systemic therapy, CN can be categorized as immediate CN (surgery first, followed by systemic therapy) or delayed CN (systemic therapy first, followed by surgery). A phase III randomized controlled trial (SURTIME) comparing immediate versus delayed CN in mRCC patients was terminated early due to slow accrual (21). However, an intention-to-treat analysis of the enrolled patients found that those receiving preoperative sunitinib followed by delayed CN had a significantly longer median overall survival than those undergoing immediate surgery (32.4 months vs. 15.0 months; HR = 0.57, p=0.03). This finding is supported by subsequent analyses of the CARMENA trial data, which identified a subset of patients in the sunitinib monotherapy arm who subsequently underwent delayed CN, suggesting a rationale for this sequential approach (7, 22, 23). Furthermore, in a comprehensive systematic review, Bhindi et al. reported that delayed CN following targeted therapy did not increase perioperative complication rates (Clavien-Dindo ≥3 complications: 3%-29%) and allowed clinicians to identify patients most likely to benefit from surgery, thereby improving overall survival (8). A multivariate analysis by the IMDC of mRCC patients receiving ICIs or TKIs showed that within the ICI subgroup, patients who underwent CN had a 39% reduced risk of death (HR = 0.61, p = 0.013), indicating potential survival benefit for CN even in the immunotherapy era (24). Consistent with this, European systematic reviews emphasize that delayed CN following a good response to systemic therapy is a rational and safe treatment paradigm (4). In our case, treatment with toripalimab plus axitinib resulted in significant tumor shrinkage and necrosis, allowing for the safe performance of a robot-assisted radical nephrectomy. Although RECIST 1.1 categorized the radiographic change as SD, the pronounced intratumoral necrosis on imaging and the predominantly necrotic pathology suggest a meaningful biological response that may not be fully captured by size-based criteria alone. This discordance between limited size reduction and marked necrosis is clinically relevant, as it implies that neoadjuvant therapy may induce meaningful biological effects, such as tumor cell death and remodeling of stress-response pathways [like autophagy and mitochondrial dynamics, which have been linked to drug sensitivity in experimental cancer models (25)]. Therefore, size-based criteria alone may underestimate treatment activity in selected patients, and integrating necrosis-related imaging findings and pathologic response may provide a more comprehensive evaluation of neoadjuvant benefit. These findings collectively support a “systemic therapy first, surgery later” sequential strategy, particularly for patients with good performance status, controlled metastatic burden, and potentially resectable primary tumors. Based on the results of the CARMENA study and multiple retrospective analyses, some guidelines and consensuses suggest that cytoreductive nephrectomy combined with targeted agents remains a viable option under at least the following five conditions: ① Good performance status (ECOG <2); ② Absence of or minimal systemic symptoms; ③ Low metastatic burden or where surgery can debulk the majority of the overall tumor burden; ④ MSKCC or IMDC favorable-risk patients; ⑤ Severe local symptoms caused by the renal tumor. While the patient in this report met conditions ③ and ⑤, they did not meet conditions ①, ②, and ④ (being IMDC poor-risk). Therefore, this case provides a basis for potentially broadening the indications for this neoadjuvant treatment approach (4, 7, 23, 26).

In the clinical application of delayed CN, another key purpose of neoadjuvant therapy is to reduce surgical technical difficulty by downsizing tumors and alleviating local invasiveness, thereby decreasing the incidence of perioperative complications. Bhindi et al.’s systematic review (Eur Urol 2018) analyzed 29 studies involving 2,845 mRCC patients and found that neoadjuvant targeted therapy reduced primary tumor volume by a median of 31-47%, alleviating local invasiveness and reducing the rate of vascular/organ adhesion during surgery (from 41% in upfront CN to 23% in delayed CN), simplifying surgical dissection (9). Regarding key perioperative complications, relevant studies have also provided clear evidence: For bleeding risk, the SURTIME trial reported a median intraoperative blood loss of 200 mL in the delayed CN group (neoadjuvant sunitinib), which was significantly lower than the 350 mL in the upfront CN group (p=0.02) (21). Consistently, Bakouny et al.’s IMDC database analysis showed that neoadjuvant ICI-TKI combination therapy reduced the risk of grade≥3 bleeding by 42% (HR = 0.58, 95%CI: 0.36-0.94) (24). For infection and anastomotic leakage-related risks, the JAVELIN Renal 101 study noted that neoadjuvant avelumab plus axitinib did not increase the incidence of surgical site infection (8.3% vs. 9.1% in the upfront CN group) or anastomotic leakage (1.2% vs. 1.5% in the upfront CN group), a result likely attributed to reduced tissue trauma from tumor shrinkage (15). We have correlated the literature with our patient’s clinical data: the patient’s tumor shrinkage (from 107×135×133 mm to 90×77×98 mm) was accompanied by resolved surrounding inflammation, intraoperative blood loss was 200 mL (consistent with the delayed CN group in SURTIME), no organ injury occurred (only minor colon adhesion without invasion), and the postoperative wound fat liquefaction (grade 1 complication) resolved with conservative treatment—aligning with the literature’s conclusion that neoadjuvant ICI-TKI improves perioperative safety. It should only be noted that certain treatment regimens (such as preoperative treatment based on bevacizumab) may increase wound-related complications (9). Therefore, provided that appropriate multidisciplinary management and monitoring are in place, this regimen appears to be a safe and feasible perioperative option.

However, the optimal endpoint for preoperative neoadjuvant therapy warrants further discussion. In this case, the patient experienced a significant drop in hemoglobin during neoadjuvant treatment. This was likely multifactorial, related to the large primary tumor burden impairing renal erythropoietin synthesis, tumor-related chronic consumption/nutritional deficits, and the potential contribution of axitinib (a VEGFR-TKI), which might affect the bone marrow microenvironment, inhibit erythropoiesis, or increase the risk of minor occult bleeding. As the cause could not be definitively excluded, the targeted-immunotherapy combination was discontinued, and surgery was scheduled. However, fortunately, there were no severe hemorrhagic complications such as hemorrhagic shock during the perioperative period. Additionally, intraoperatively, dense adhesions between the primary tumor and the mesentery/colon complicated meticulous dissection and raised suspicion of bowel invasion. Although postoperative pathology of the resected colonic tissue revealed no malignancy (showing only fibroadipose and smooth muscle tissue with histiocytic reaction), it is plausible that the tumor response to neoadjuvant therapy induced fibrosis and tissue hardening, increasing adhesion severity. This potentially heightened the difficulty of achieving complete resection, along with the risks of intraoperative bleeding, surgical site infection, and prolonged operative time. From the safety and efficacy perspective, based on the latest international guidelines and relevant clinical evidence from our current case, we have clarified the contraindications of this sequential strategy in specific patient populations as follows:①Patients with severe underlying diseases that render them unable to tolerate neoadjuvant therapy or surgery (such as, ECOG ≥2 with frailty) (4, 7);②Active bleeding disorders or coagulopathy(neoadjuvant VEGFR-TKI therapy may increase the risk of severe hemorrhage) (9);③Patients with progressive infections (for instance, sepsis), uncontrolled autoimmune diseases or transplant-related concerns (8);④Rapidly progressive disease on induction therapy or lack of early clinical benefit;⑤High surgical/anatomic risk where neoadjuvant therapy is unlikely to reduce complexity (for instance, uncontrolled bleeding risk, unresectable local invasion, very high-level IVC-TT requiring urgent thrombectomy) (4);⑥Contraindications to VEGFR-TKIs (uncontrolled hypertension, significant proteinuria, recent major bleeding, or non-healed wounds) (8, 14, 15).

Given the dynamic and context-dependent nature of immune microenvironments, patient selection for neoadjuvant immunotherapy-based strategies should remain individualized rather than ‘one-size-fits-all’ (27). Recent studies have shown that tumor-educated stromal and myeloid populations can synergistically regulate immune suppression (28), and the activation of some cytokine-related signaling pathways (such as the IL-6/STAT3 axis) is widely associated with tumor progression and resistance to immunotherapy (29). This also highlights the necessity of using biomarkers to guide patient stratification in perioperative combination therapy strategies, which supports the need for careful patient stratification when adopting sequential strategies. For specific patient populations, this sequential strategy should be accompanied by an explicit discussion of differential applicability and patient stratification, because the benefit of surgery depends strongly on metastatic pattern, risk category, and local anatomic complexity. In patients with venous tumor thrombus (VTT), nephrectomy with thrombectomy is inherently high-risk and should be planned in a multidisciplinary setting (7). Early neoadjuvant evidence suggests axitinib-based regimens may downstage thrombus height without increasing Mayo level (NEOTAX: 44% downstaging; NAXIVA: 35% Mayo-level reduction), potentially facilitating surgery (30, 31);however, rare events such as thrombus fragmentation or bleeding remain insufficiently characterized. Close imaging surveillance and strict perioperative management are therefore essential, including holding axitinib around surgery (at least 2 days preoperatively and restarting no earlier than 2 weeks after major surgery once wound healing is adequate).Metastatic pattern and burden should guide sequencing. Current guidelines prioritize systemic therapy for stage IV disease and reserve CN for carefully selected responders with good performance status and limited, resectable disease (for example, nodal-only or oligometastatic disease) (7); patients with high-volume multi-organ metastases or rapid progression are less likely to benefit and should generally continue systemic therapy (4). Based on these guideline principles, we added a pragmatic stratification statement: patients with nodal-only/oligometastatic disease (where most tumor burden can be removed) may be considered for delayed CN after documented response, whereas patients with high-volume multi-organ metastases (often including liver/bone/brain) are less likely to benefit from surgery and should prioritize systemic therapy.

Nevertheless, the follow-up period of the reported patient was excessively short at the time of manuscript submission. So we have formalized a 3-year long-term follow-up protocol for the patient, with scheduled assessments at 3, 6, 12, 24, and 36 months postoperatively. We will meticulously record data on disease progression, recurrence, adverse events (32), and quality of life. We plan to initiate a small-scale prospective observational study, aiming to recruit 15–20 patients with advanced ccRCC in the future. The primary endpoints are the 12-month progression-free survival rate and the 24-month overall survival rate, in order to verify the long-term survival benefits of this sequential strategy (33).In addition, we will conduct single-cell analysis, proteomics, genomics, transcriptomics, metagenomics, and metabolomics studies on blood, urine, feces, and tumor specimens obtained from surgeries of these patient. On the one hand, this helps identify predictive biomarkers and integrate clinicopathological and sequencing work to continuously refine the taxonomy of renal neoplasms for personalized treatment (34, 35). On the other hand, radiomics can be combined with transcriptomic features for prognostic prediction of aRCC/mRCC (36), which provides support for future efforts to improve perioperative efficacy evaluation. Moreover, current clinical prognostic models for advanced RCC-such as the IMDC risk classification-remain imperfect in capturing the biological heterogeneity of ccRCC and may be insufficient to support truly individualized perioperative decision-making after patient stratification. Given this heterogeneity, emerging molecular parameters such as tumor-normal mitochondrial DNA (mtDNA) copy-number enrichment/variation have been proposed as independent prognostic factors that may complement existing clinical risk models and, in future studies, help refine candidate selection for neoadjuvant ICI-TKI therapy followed by deferred CN (37). In addition, microorganisms in urine and feces, as well as intratumoral flora in renal tumors, can also be used to study the regulation of tumor immunity and the synergistic effect with PD-1 inhibitors (38). The complications after CN are also worthy of great attention, such as the incidence rate, risk factors of postoperative venous thromboembolism, and the role of anticoagulant therapy. Beyond oncologic control, supportive care remains important during systemic therapy and postoperative recovery. An evidence map of systematic reviews suggests that acupuncture and moxibustion have been reported to alleviate several cancer-related conditions (like cancer-related fatigue, nausea/vomiting, and pain) (39). Given that postoperative incisional fat liquefaction has been observed in our patients, adjuvant strategies to promote wound repair deserve further research. In a rat pressure-ulcer model, controlled-temperature moxibustion accelerated wound healing and reduced inflammatory signaling and apoptosis, potentially through modulation of the PI3K/AKT/mTOR pathway (40); whether this effect can be translated and applied to postoperative incisional complications in neoadjuvant therapy for renal cell carcinoma remains to be verified by clinical studies.

On the whole, neoadjuvant therapy with axitinib combined with toripalimab induced significant tumor shrinkage and necrosis in this patient, creating favorable conditions for a subsequent delayed cytoreductive nephrectomy. The encouraging clinical efficacy and acceptable safety profile observed in this case support the feasibility of combining immune-targeted therapy with delayed CN in selected aRCC/mRCC patients.

## Conclusion

In the era of combination targeted therapy and immunotherapy, the role of deferred CN is being redefined in aRCC/mRCC. While neoadjuvant therapy or delayed CN may offer survival benefits in carefully selected patients, there is currently a lack of high-level evidence supporting neoadjuvant treatment strategies. Although there is currently no strong evidence to support neoadjuvant therapy, considering the theoretical advantages of neoadjuvant immunotherapy—it may generate a stronger and more durable anti-tumor immune response and may shrink the primary tumor—neoadjuvant therapy and perioperative therapy require long-term follow-up of patients’ prognosis in clinical trials. Drawing on research experience gained in other types of urinary tumors (41), it is necessary to conduct randomized trials comparing with the standard treatment regimen, as well as clinical trials to develop individualized debulking strategies for specific patient populations. Renal cell carcinoma (RCC) lacks biomarkers that aid in patient selection and treatment debulking, and regarding the interactions between different cell types in the tumor microenvironment of aRCC/mRCC, translational research should be encouraged.

Furthermore, safety aspects require further investigation. Although the present case demonstrated good tolerability of neoadjuvant axitinib combined with toripalimab, potential risks remain, including hematological toxicity, local tissue fibrosis, and intraoperative complications. Future research should focus on evaluating the short- and long-term safety of this sequential treatment regimen, particularly the effects of the drugs on bone marrow hematopoiesis, the coagulation system, and tissue repair. Concurrently, it is necessary to establish standardized perioperative monitoring and management protocols to optimize the timing of drug discontinuation and surgery. This will help minimize the risks associated with immune- and targeted therapy-related adverse events, thereby ensuring overall treatment safety and controllability.

## Data Availability

The raw data supporting the conclusions of this article will be made available by the authors, without undue reservation.
